# Blast vacuolization in AML patients indicates adverse-risk AML and is associated with impaired survival after intensive induction chemotherapy

**DOI:** 10.1371/journal.pone.0223013

**Published:** 2019-09-30

**Authors:** Olivier Ballo, Jan Stratmann, Hubert Serve, Björn Steffen, Fabian Finkelmeier, Christian Brandts

**Affiliations:** 1 Department of Medicine, Hematology/Oncology, Goethe University, Theodor-Stern-Kai, Frankfurt/Main, Germany; 2 German Cancer Consortium (DKTK) and German Cancer Research Center (DKFZ), Heidelberg, Germany; 3 Department of Medicine, Gastroenterology, Hepatology and Endocrinology, Goethe University, Theodor-Stern-Kai, Frankfurt/Main, Germany; European Institute of Oncology, ITALY

## Abstract

**Introduction:**

Vacuolization is a frequently found morphological feature in acute myeloid leukemia (AML) blasts. Subcellular origin and biological function as well as prognostic impact are currently unknown. The aim of this study was to evaluate whether vacuolization correlates with clinically relevant AML features.

**Materials & methods:**

Bone marrow smears of patients diagnosed with AML at the University Hospital Frankfurt between January 2011 and August 2013 were analyzed for blast vacuolization and correlated with clinically relevant AML features. Patients undergoing standard induction chemotherapy were further analyzed for molecular and cytogenetic features as well as treatment response and survival.

**Results:**

14 of 100 patients diagnosed with AML receiving standard induction chemotherapy had evidence of blast vacuolization. Positivity for vacuolization correlated with a CD15 positive immunophenotype and with a higher incidence of high-risk AML according to the European LeukemiaNet risk stratification. AML patients with blast vacuolization had a poor blast clearance after standard induction chemotherapy and poor survival.

**Discussion:**

In conclusion, our findings demonstrate that vacuolization can easily be determined in myeloid leukemia blasts and may be a useful biomarker to predict AML risk groups as well as early treatment response rates and survival.

## Introduction

Light microscopy examinations of peripheral blood and bone marrow smears have been the foundation for diagnosis of acute leukemia, since it was first described by Rudolf Virchow in 1845 [1]. In case of acute myeloid leukemia (AML) unique morphological characteristics such as Auer rods or hypergranulation allow subclassification according to the French-American-British classification system (FAB) [2]. These morphological features have impact on prognosis, further diagnostic steps and initiation of therapy. For instance, the acute promyelocytic leukemia (APL) with the classical M3 morphology needs to be confirmed by polymerase chain reaction (PCR) or fluorescent in-situ hybridization (FISH) for PML/RARA fusion gene and is treated with an *all-trans retinoic acid* based therapy [3], AML-M4Eo with inv(16)(p13q22) on the other hand is characterized by bone marrow eosinophilia and has a favorable prognosis [4].

We observed blast vacuolization in a subset of AML patients. A literature review revealed only limited information about the frequency and no information about the biological function or the clinical significance of blast vacuolization in AML patients. Gervais et al. analyzed 33 AML patients with MYST histone acetyltransferase 3 (MYST3) rearrangements for its most relevant characteristics. Cytoplasmic vacuoles were noted in 21 of these cases (no pictures) with no further information [5]. Studies with pictures of blast vacuolization in bone marrow smears of AML patients have been published by Tang et al. in myeloid neoplasms (AML and MDS) with MLL gene amplification [6] and twice in AML patients with tetraploidy or near-tetraploidy, again with no further information [7, 8]. In contrast, in the context of acute lymphatic leukemia (ALL) the extent of vacuolization is critical for determining the FAB subclassification [2]. A typical ALL-L3 morphology—large varied cells with vacuoles - needs to be confirmed by further immunophenotypic analysis, since the discrimination between mature B-ALL (Burkitt lymphoma/leukemia) and Pro-B-ALL has impact on the choice of therapy and prognosis [9, 10].

Although light microscopy has been an established diagnostic tool for decades in diagnosing AML, to our knowledge there are no reports about the clinical role or biological function of vacuoles in myeloid blasts. In this work we evaluated the association of positivity for vacuolization in AML blasts with molecular and cytogenetic AML features, as well as with early treatment response and survival rates.

## Study design and methods

### Patient selection and diagnostic workup

In this retrospective single center study bone marrow smears from AML patients diagnosed at the University Hospital Frankfurt between January 2011 and August 2013 were analyzed for vacuolization. After bone marrow puncture at time of diagnosis bone marrow smears were prepared using May-Grünwald-Giemsa staining [11]. Two bone marrow slides of each AML patient were retrospectively screened for blast vacuolization. Two independent investigators confirmed positivity for AML blast vacuolization. The quantity of vacuoles for each of 100 AML blasts was separately documented for all patients. We defined AML blasts to be positive for vacuolization if more than 10% of AML blasts had at least one vacuole. Bone marrow slides were viewed with a Zeiss Axioskop 2 plus light microscope using a 63x Zeiss oil immersion objective. Images were captured by a Sony Exwave HAD CCD camera and exported as image-files using Dietermann & Heuser Solution GmbH Image Data Base (dhs Image Data Base). For better comparability of response rates and outcome only patients qualifying for intensive induction chemotherapy were further investigated. Molecular testing for NPM1, MLL, FLT3-ITD and FLT3-TKD as well as cytogenetic analysis was performed by MLL Munich Leukemia Laboratory, Germany. Antibodies for fluorescence-activated cell sorting (FACS) were provided by BD biosciences. FACSCanto II (BD Biosciences) was used for cell sorting. In this work a marker expression in immunophenotyping was considered positive if >20% of the cells in the blast gate were positive for the tested marker. Response criteria and determination of genetic risk group were defined as recommended from the international expert panel on behalf of the European LeukemiaNet [12]. Blast clearance was achieved if <10% of myeloid blasts persisted in cytological examination of day 15 bone marrow evaluation after intensive induction chemotherapy [13]. The study was performed in accordance with the 2013 Helsinki declaration. Patients were enrolled in the AML registry of the Study Alliance Leukemia (EK 98032010) and/or in the clinical cancer registry of the University Cancer Center (UCT) Frankfurt. Patients provided informed written consent to have data from their medical records used anonymously in research. The local ethical committee of the University Hospital Frankfurt approved the study.

### Clinical features

Standard intensive induction chemotherapy was the so-called *7+3*-regime; cytarabine 100mg/m^2^ given intravenous (IV) continuously for 7 days is combined with daunorubicin 60mg/m^2^ given as a 30minute IV infusion on days 3, 4 and 5 [14]. Patients under the age of 60 received a second induction therapy with *7+3* if early blast clearance was achieved on day 15 bone marrow evaluation or with high-dose cytarabine using the *HAM protocol* (cytarabine 3000mg/m^2^ was administered by 3-hour IV infusion every 12 hours on day 1 through 3 and mitoxantrone 10mg/m^2^ by 30-minute IV infusion on day 3,4 and 5) if blast clearance was not achieved on day 15 bone marrow evaluation [15]. Patients above the age of 60 received only a second induction chemotherapy with HAM (with reduced cytarabine dose of 1000mg/m^2^), if the first induction chemotherapy cycle was not sufficient to achieve bone marrow blast clearance on day 15 [16]. Poor response on day 15 bone marrow evaluation was defined as >10% blasts persisting for patients under the age of 60, for patients above the age of 60 poor response on day 15 was defined as >5% blasts persisting [13].

### Statistical analysis

This study was designed as a retrospective cohort study. Patients were followed till death or last contact. Continuous variables are shown as median ± range and categorical variables are reported as frequencies and percentages. All continuous variables were tested for normality and were analyzed by using the Student´s *t*-test or the Wilcoxon-Mann-Whitney test accordingly. Fishers exact test was used for binary variables. Death rates were analyzed by Kaplan-Meier method and curves were compared by log-rank test. For assessment of independent predictors of blast clearance on day 15 bone marrow evaluation, a binary logistic regression was used. For assessment of independent predictors of survival, a multivariate Cox regression hazard model with forward stepwise (likelihood ratio) entry was used, factors with p<0.1 in the univariate analysis were included. Statistical analysis was performed with SPSS (Version 22.0, IBM, Armonk, NY).

## Results

In this retrospective single center study we analyzed bone marrow smears from 134 AML patients diagnosed at the University Hospital Frankfurt between January 2011 and August 2013 for vacuolization. Vacuolization of AML blasts was found in 16 of 134 AML patients (11.9%) while 118 patients had AML without blast vacuolization. 34 patients went on to receive a non-intensive chemotherapy due to advanced age or bad performance status. After exclusion of those patients, 14 patients (14%) with vacuolized AML blasts and 86 patients (86%) with nonvacuolized AML blasts were further investigated.

### Clinical features

Median age was 64.5 years (range 34–74) in patients with vacuolized AML blasts and 58.5 years (range 18–81) in AML patients without evidence of blast vacuolization (p = 0.344). At time of diagnosis there was no significant difference between the two groups with respect to gender, lactate dehydrogenase (LDH), WHO classification, peripheral blood cell counts or blast count in the bone marrow or in the peripheral blood. (**Table 1**).

**Table 1:** Baseline characteristics.

Characteristic	All patients	vAML	non-vAML	*p* Value
Number of patients (n, %)	100 (100)	14 (14)	86 (86)	
Median age (median, range)	59 (18–81)	64.5 (34–74)	58.5 (18–81)	0.344
Male sex (n, %)	45 (45)	4 (28.6)	41 (47.7)	0.250
Leukocytes/nl* (median, range)	13.8 (0.31–300)	20 (1.84–122)	12.75 (0.31–300)	0.772
Hemoglobin g/dl* (median, range)	8.9 (4.2–18.5)	8.55 (4.6–12.7)	9 (4.2–18.5)	0.387
Thrombocytes/nl* (median, range)	50 (6–432)	55 (11–228)	50 (6–432)	0.323
Lactate dehydrogenase U/l* (median, range)	390 (139–4700)	567 (217–1763)	344 (139–4700)	0.216
Peripheral blood blast count* (median, range)	26 (0–92)	12 (0–92)	28 (0–87)	0.386
Bone marrow blast count* (median, range)	52 (6–95)	51 (17–95)	56 (6–90)	0.386
AML with t(8;21)(q22;q22.1) (n, %)	2 (2)	0 (0)	2 (2.3)	1.000
AML with inv(16)(p13.1q22) or t(16;16)(p13.1;q22) (n, %)	2 (2)	1 (7.1)	1 (1.2)	0.262
AML with t(9;11)(p21.3;q23.3) (n, %)	1 (1)	0 (0)	1 (1.2)	1.000
AML with inv(3)(q21.3q26.2) or t(3;3)(q21.3;q26.2) (n, %)	1 (1)	1 (7.1)	0 (0)	0.140
AML with BCR-ABL1 (n, %)	1 (1)	0 (0)	1 (1.2)	1.000
AML with mutated NPM1 (n, %)	32 (32)	3 (21.4)	29 (33.7)	0.377
AML with mutated RUNX1 (n, %)	2 (2)	1 (7.1)	1 (1.2)	0.262
AML with myelodysplasia-related changes (n, %)	31 (31)	6 (42.9)	25 (29.0)	0.354
Therapy-related myeloid neoplasms (n, %)	3 (3)	1 (7.1)	2 (2.3)	0.367
AML not otherwise specified (n, %)	25 (25)	1 (7.1)	24 (27.9)	0.179
AML with myelomonocytic differentiation (n, %)	54 (54)	8 (57.1)	46 (53.5)	1.000

*at time of diagnosis. All p-values reported are two-sided. Statistical significance was defined as p≤0.05. vAML indicates patients with vacuolization of AML blasts, non-vAML indicates patients without signs of blast vacuolization.

### Cytological features

**Fig 1:**
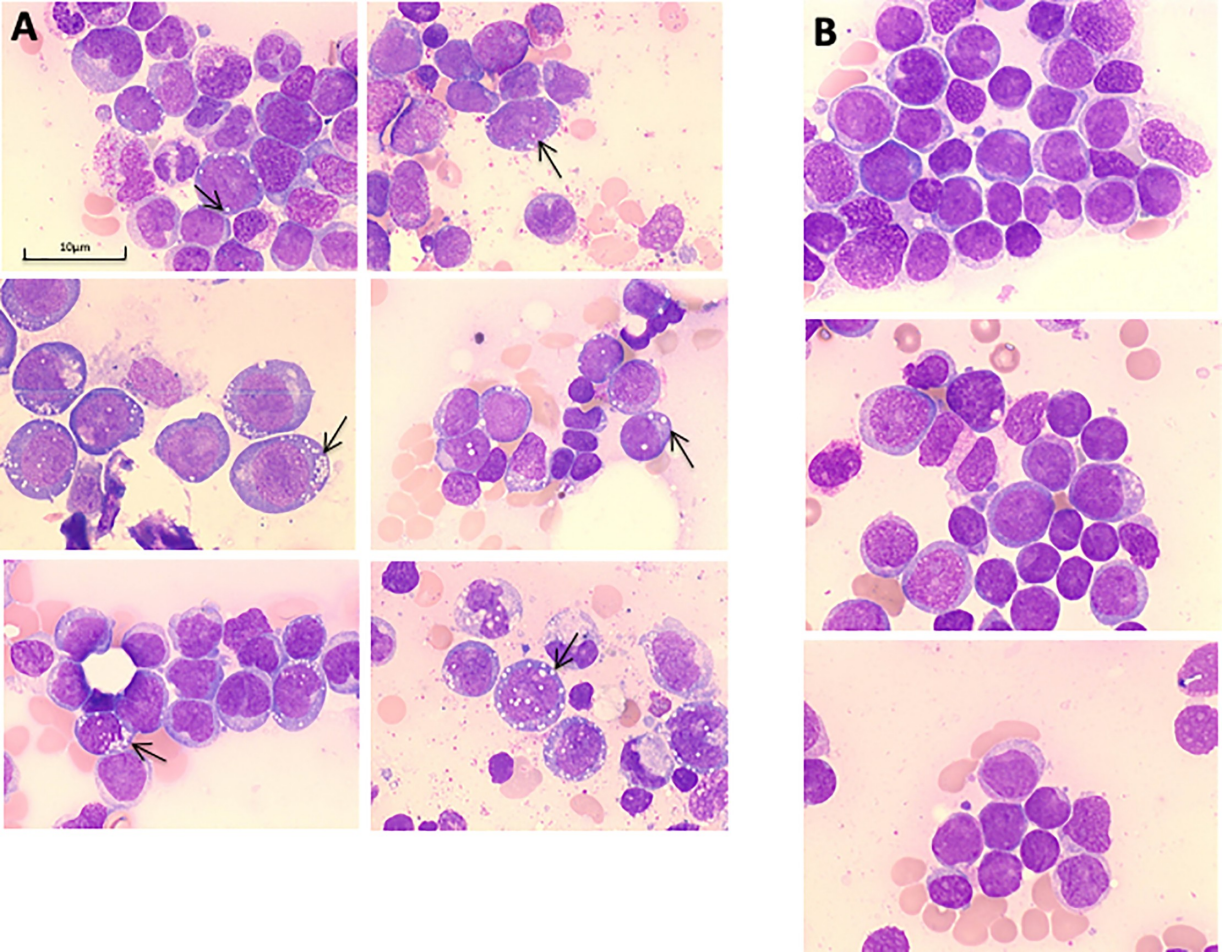
**A.** Typical morphology of bone marrow smears in Pappenheim staining from 6 individual AML patients with blast vacuolization. **B.** Typical morphology of bone marrow smears in Pappenheim staining from 3 regular AML patients without signs of blast vacuolization.

The vacuoles seen in AML blasts localize mostly in the cytoplasma, rarely vacuoles project above the nucleus (**Fig 1A**). The appearance can range from approximately 50 to over 1000 vacuoles per 100 AML blasts. The distribution within AML blasts can differ from few single vacuoles per blast cell to groups of >35 vacuoles vacuoles per blast cell. The median percentage of blasts with at least one vacuole was 0% (range 0–8) in AML patients without blast vacuolization and 29% (range 11–91) in AML patients with blast vacuolization. The size of a vacuole ranges from 0.5–2.0 μm. In AML patients without vacuolization generally no vacuoles can be found in any views of different bone marrow smears (**Fig 1B**).

### Immunophenotypic analysis

No significant difference was seen in the expression of the myeloid markers (CD13, CD33, MPO), the progenitor cell markers (CD34, CD117) or the lymphatic markers (CD3, CD10, CD19, HLA-DR) between patients with and without vacuolized AML blasts (**Table 2**). However all AML patients with blast vacuolization who were tested for CD15 expression were positive (100% vs. 55.2%, p = 0.01).

**Table 2:** Immunophenotypic analysis of the bone marrow.

Characteristic	vAML	non-vAML	*p* Value
Number of patients (n, %)	14	86	** **
CD34+ (%, tested patients)	70 (10)	54.0 (63)	0.497
CD15+ (%, tested patients)	100 (10)	55.2 (58)	0.010
MPO+ (%, tested patients)	70 (10)	63.2 (58)	1.0
CD117+ (%, tested patients)	72.7 (11)	78.6 (70)	0.701
CD13+ (%, tested patients)	100 (11)	90.1 (76)	0.588
CD11b+ (%, tested patients)	85.7 (7)	50 (46)	0.112
CD33+ (%, tested patients)	100 (10)	93.5 (77)	1.0
CD11c+ (%, tested patients)	100 (10)	94.9 (78)	0.341

All p-values reported are two-sided. Statistical significance was defined as p≤0.05. vAML indicates patients with vacuolization of AML blasts, non-vAML indicates patients without signs of blast vacuolization

### Cytogenetic and molecular analysis

Molecular aberrations were equally distributed between patients with and without vacuolized AML blasts (**Table 3**). Cytogenetic aberrations were found in 8 patients with vacuolized AML blasts and in 32 AML patients without blast vacuolization (n = 8, 57.1% vs. n = 32, 37.2%, p = 0.244). A significantly higher incidence of adverse-risk AML was found in AML patients with blast vacuolization when cytogenetic and molecular genetic data was combined into genetic risk groups according to the 2010 ELN guidelines (n = 8, 57.1% vs. n = 22, 25.6%, p = 0.026) [12]. Detailed information about the cytogenetic and molecular alterations found in AML patients with blast vacuolization are listed in **Table 4**.

**Table 3:** Molecular and cytogenetic analysis.

Characteristic	vAML	non-vAML	*p* Value
Number of patients (n, %)	14 (14)	86 (86)	
Adverse-risk AML (n, %)	7 (50)	16 (18.6)	0.016
Abnormal karyotype (n, %)	8 (57.1)	32 (37.2)	0.244
NPM1 (n, %)	3 (21.4)	30 (34.9)	0.377
FLT3-ITD (n, %)	3 (23.1)	19 (21.8)	1.0
FLT3-TKD (n, %)	1 (7.1)	6 (6.98)	1.0
MLL rearrangements (n, %)	0 (0)	6 (6.98)	0.591

All p-values reported are two-sided. Statistical significance was defined as p≤0.05. vAML indicates patients with vacuolization of AML blasts, non-vAML indicates patients without signs of blast vacuolization.

**Table 4:** Cytogenetic und molecular information in AML patients with blast vacuolization.

Patient ID	Karyotype	Mutations
1	46, XY, del(5)(q14q34), der(7)t(7;21)(q11;q11), +8, -18, del(21)(q11q22)	No mutation
2	Complex karyotype with del5	No mutation
3	46, XX	FLT3-ITD+NPM1 mutation
4	Complex karyotype with involvement of chromosome 2, 3, 4, 7, 9, 10, 12, 14, 15, 16, 17. In 2 metaphases deletion in long of chromosome 5	No mutation
5	46, XX	2 RUNX1 gene mutations, D816V mutation of the KIT gene
6	46, XY	No mutation
7	46, XY, inv(16)(p13q22) CBFB/MYH11	No mutation
8	46, XX	FLT3-ITD mutation
9	46, XX, inv(3)(q21q26)	No mutation
10	46, XY	FLT3-ITD+NPM1 mutation
11	Complex karyotype 45, XX, der(3)t(3;10)(p25;p11), der(7)t(7;9)(q31;q22), der(9)t(9;11)(q22;q13), dic(10;11)(p11;q11)(9)	No mutation
12	Complex karyotype, 5q31-Deletion, 5q33-Deletion, 1p32-Signal, 7q31-Deletion, TP53-Deletion, BCL2-Deletion	No mutation
13	Complex karyotype	No mutation
14	46, XX	FLT3-TKD+NPM1 mutation

### Response rates to induction chemotherapy

Day 15 bone marrow evaluation was performed for 12 of the AML patients being positive for vacuolization and in 81 of the other AML patients. Missing day 15 bone marrow evaluation (7 patients) was due to death (4 patients), due to bone marrow smears with insufficient quality for cytological examination (1 patient), due to refusal of consent for performance of bone marrow aspiration (1 patient) and in one patient day 15 bone marrow evaluation was not performed due to treatment on intensive care unit for myocardial infarction. Patients with vacuolized AML blasts had a significantly worse blast clearance rate on day 15 after induction chemotherapy (n = 2, 16.7% vs. n = 47, 54.6%, p = 0.011) (**Table 5**). Consequently, only for two (22.2%) of the patients with vacuolized AML blasts a standard induction chemotherapy with the *7+3* protocol was sufficient to achieve a complete remission (CR), whereas for 40 (64.5%) of the AML patients without blast vacuolization the 7+3 protocol was sufficient for CR achievement (p = 0.0289).

**Table 5:** Clinical findings.

Characteristic	vAML	non-vAML	*p* Value
Number of patients (n, %)	14 (14)	86 (86)	** **
Day 15 bone marrow blast clearance (n, %)	2 (16.7)	47 (54.6)	0.011
Single induction chemotherapy (n, %)	7 (50)	40 (46.5)	1.0
Double induction chemotherapy (n, %)	7 (50)	46 (53.5)	1.0
Allogenic stem cell transplantation as consolidation therapy (n, %)	6 (42.9)	41 (47.7)	0.775
Complete remission after induction chemotherapy (n, %)	9 (69.3)	62 (71.3)	1.0
Complete remission after induction chemotherapy with *7+3* (n, %)	2 (22.2%)	40 (64.5)	0.0289

All p-values reported are two-sided. Statistical significance was defined as p≤0.05. vAML indicates patients with vacuolization of AML blasts, non-vAML indicates patients without signs of blast vacuolization.

Those patients not achieving a blast clearance on d15 went on for a second more intensive induction chemotherapy (HAM) to salvage for a CR. Cytological CR rates after completion of induction chemotherapy were equal between the two groups (n = 9, 69.3% vs. n = 62, 71.3%, p = 1.0).

### Blast vacuolization as a risk factor for incomplete blast clearance on day 15 bone marrow evaluation and survival

To further analyze vacuolization of AML blasts as a predictor for incomplete blast clearance on day 15 bone marrow evaluation a binary logistic regression model with forward stepwise likelihood ratio was performed. The nominal dichotome variables, age above 60, gender, vacuolization of AML blasts, abnormal karyotype and adverse-risk AML were included in this model. As shown in **Table 6**vacuolization of AML blasts was an independent risk factor for poor blast clearance on day 15 bone marrow evaluation after induction chemotherapy (odds ratio (OR) = 8.042, 95% confidence interval (CI) 1.590–40.692, p = 0.012).

**Table 6:** Logistic regression analysis of risk factors for failure of day 15 bone marrow blast clearance.

Parameter	OR	95% CI	*P* value	OR	95% CI	*P* value
	Univariate analysis			Multivariate analysis		
Age > 60	2.186	0.945–5.056	0.068	3.064	1.177–7.978	0.022
Male sex	1.324	0.582–3.010	0.503			
Adverse-risk AML	3.429	1.250–9.402	0.017	3.408	1.095–10.607	0.034
Abnormal karyotype	2.062	0.895–4.748	0.089			
Vacuolization of AML blasts	6.912	1.422–33.590	0.017	6.671	1.198–37.147	0.003

Risk factors for day 15 response were determined using a uni- and multivariate binary logistic regression model. All p-values reported are two-sided. Statistical significance was defined as p≤0.05.

**Fig 2:**
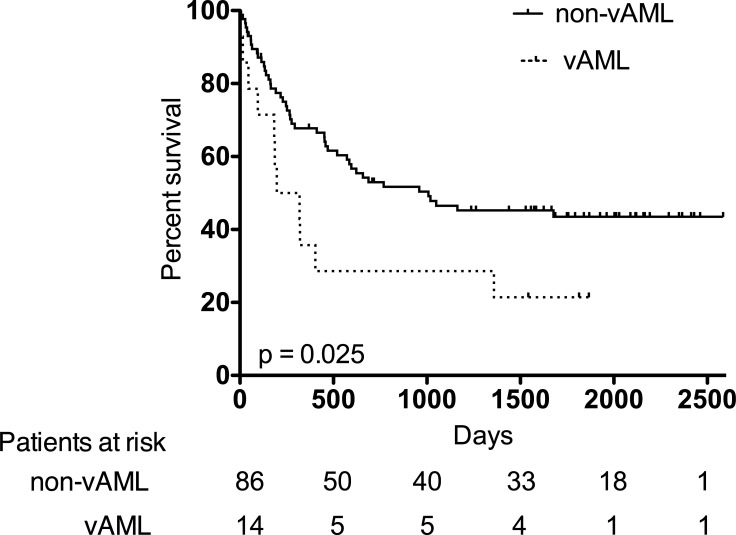
Kaplan-Meier curves for overall survival (OS). Dotted line indicates OS of AML patients with blast vacuolization (vAML) and solid line indicates OS of AML patients without evidence of blast vacuolization (non-vAML).

We analyzed the survival of both patient cohorts. Kaplan-Meier estimates for OS are displayed in **Fig 2**. Here, AML patients with blast vacuolization showed a poor survival rate. To further analyze blast vacuolization as a prognostic parameter in AML patients undergoing intensive induction chemotherapy a multivariate Cox regression model with forward stepwise likelihood ratio was performed. The nominal dichotome variables age above 60 years, male sex, adverse-risk AML, allogenic SCT as consolidation therapy, CR after induction chemotherapy and vacuolization of AML blasts were included in this model. As shown in **Table 7**age above 60 years, allogenic SCT as consolidation therapy, CR after induction chemotherapy and vacuolization of AML blasts were independently associated with OS.

**Table 7:** Univariate and multivariate analysis associated with survival in AML patients.

Parameter	HR	95% CI	*P* value	HR	95% CI	*P* value
	Univariate analysis			Multivariate analysis		
Age > 60	3.338	1.938–5.750	<0.001	2.140	1.184–3.870	<0.001
Male sex	1.300	0.767–2.202	0.330			
Adverse-risk AML	1.962	1.118–3.442	0.019			
Complete remission after induction chemotherapy	0.276	0.162–0.470	<0.001	0.322	0.186–0.559	<0.001
Stem cell transplantation as consolidation therapy	0.349	0.202–0.604	<0.001	0.428	0.236–0.777	0.005
Vacuolization of AML blasts	1.992	1.029–3.855	0.041	2.085	1.057–4.112	0.034

CI indicates confidence interval and HR hazard ratio. All p-values reported are two-sided. Statistical significance was defined as p≤0.05

## Discussion

During our routinely cytological diagnostic we repeatedly noticed the appearance of vacuoles in myeloid leukemia blasts. We found description of this morphological feature in some but not all reports written by the examining cytologist when diagnosing AML. Our literature review revealed very limited data about the appearance of blast vacuolization in AML patients and no data about its etiology or clinical role [5–8]. To our knowledge, we here provide for the first time detailed information about vacuolization in myeloid leukemia blasts and define its relevant characteristics.

In our study vacuolization of AML blasts at diagnosis correlates with a CD15-positive immunophenotype. CD15 is a cluster of differentiation antigen mediating chemotaxis and phagocytosis [17]. Its prognostic role in leukemia patients remains controversial [18–20]. Recently Chisini *et al*. published a large retrospective analysis of 460 AML patients—173 being positive for CD15—and demonstrated CD15 to be of independent prognostic value for CR achievement. Those patients tend to have a superior survival rate, although this analysis missed statistical significance [21]. This rather favorable impact on AML is in contrast to our results indicating poor survival and poor response of AML with blast vacuolization to standard induction chemotherapy.

Recent studies identified a new cytomorphological feature in AML blasts and found a correlation of the so-called *cup-like acute myeloid leukemia* with mutations of FLT3 and NPM1 [22–24]. Thus, we were curious to see whether our cytomorphological feature also correlates with cytogenetic or molecular AML features. Our analysis showed that AML patients with blast vacuolization had a significantly increased proportion of adverse-risk AML (according to the 2010 ELN guidelines) when compared to AML patients without blast vacuolization. Notably, 6 of 7 (85.7%) adverse-risk vAML patients had myelodysplasia-related changes, whereas in the non-vAML patients 21 of 22 with adverse-risk features (95.5%) had myelodysplasia-related changes. Thus, we conclude that for both the vAML and non-vAML cohort high-risk features are ascribable to AML with myelodysplasia-related changes. The specific impact of blast vacuolization on the one hand and the impact of adverse-risk AML and AML with myelodysplasia-related changes on the other hand on poor prognosis and low rates of complete remission in vAML patients needs to be further analyzed in a larger cohort. Furthermore, a significantly larger cohort would allow analyzing the clinical course of vAML and non-vAML patients in molecular AML subgroups.

We analyzed the impact of AML blast vacuolization on day 15 blast clearance and complete remission rates after induction chemotherapy. AML patients being positive for blast vacuolization had a significantly reduced blast clearance on day 15 bone marrow evaluation and consequently a poor response rate to standard induction chemotherapy with the *7+3* protocol. However, blast persistence on day 15 was successfully salvaged with high dose cytarabine combined with mitoxantrone (HAM) also in AML patients with blast vacuolization, suggesting a sensitivity to cytarabine of the vacuolized AML subgroup. As a result both populations had equal CR rates after admission of the second induction chemotherapy cycle. Further prospective analysis will have to investigate whether an initial treatment with HAM should be favored over the standard induction chemotherapy with *7+3* in AML patients with blast vacuolization.

The Kaplan-Meier estimates for OS showed poor survival for AML patients with blast vacuolization and our multivariate analysis identified blast vacuolization to be independently associated with OS. A limitation of our analysis is the limited number of AML patients and the low percentage of patients being positive for blast vacuolization. Retrospective analyses of a larger cohorts are required to address the impact of blast vacuolization in AML patients on OS in more detail.

In conclusion, vacuolization can easily be determined in myeloid leukemia blasts by light microscopy. AML patients with blast vacuolization in bone marrow smears have reduced responsiveness to standard induction chemotherapy and impaired survival. Whether vacuoles are also found in the peripheral blood was not analyzed in our study. We hypothesize that vacuoles in AML blasts may represent dysfunctionally enlarged autophagosomes or other cellular organelles. Indeed, Chen et al. (2016) detected a similar blast vacuolization *in vitro* when treating AML U937 cell lines with lapatinib and demonstrated that these vacuoles appear with lapatinib-induced autophagic cell death [25]. Nevertheless, the blasts require a more detailed ultrastructural analysis and further studies are needed to confirm our findings in a larger cohort and to determine whether this morphological feature can be useful in clinical routine.

## Supporting information

S1 Table Raw data. (SAV)

## Abbreviations

AML: Acute myeloid leukemia
ALL: Acute lymphoblastic leukemia
APL: Acute promyelocytic leukemia
CI: Confidence interval
CR: Complete remission
FAB: French-American-British classification system
FISH: Fluorescent in-situ hybridization
HAM: High-dose cytosine arabinoside and mitoxantrone
IV: Intravenous
LDH: Lactate dehydrogenase
OR: Odds ratio
OS: Overall survival
PCR: Polymerase chain reaction
SCT: Stem cell transplantation
vAML: vacuolized acute myeloid leukemia
